# Molecular Simulation of MoS_2_ Exfoliation

**DOI:** 10.1038/s41598-018-35008-z

**Published:** 2018-11-13

**Authors:** Guoqing Zhou, Pankaj Rajak, Sandhya Susarla, Pulickel M. Ajayan, Rajiv K. Kalia, Aiichiro Nakano, Priya Vashishta

**Affiliations:** 1 Collaboratory of Advanced Computing and Simulation, Department of Physics and Astronomy, University of Southern California, Los Angeles, USA; 2 Mork Family Department of Chemical Engineering and Materials Science, University of Southern California, Los Angeles, USA; 3 Department of Computer Science, University of Southern California, Los Angeles, California 90089 USA; 4 Department of Materials Science and Nanoengineering, Rice University, Houston, Texas 77005 USA

## Abstract

A wide variety of two-dimensional layered materials are synthesized by liquid-phase exfoliation. Here we examine exfoliation of MoS_2_ into nanosheets in a mixture of water and isopropanol (IPA) containing cavitation bubbles. Using force fields optimized with experimental data on interfacial energies between MoS_2_ and the solvent, multimillion-atom molecular dynamics simulations are performed in conjunction with experiments to examine shock-induced collapse of cavitation bubbles and the resulting exfoliation of MoS_2_. The collapse of cavitation bubbles generates high-speed nanojets and shock waves in the solvent. Large shear stresses due to the nanojet impact on MoS_2_ surfaces initiate exfoliation, and shock waves reflected from MoS_2_ surfaces enhance exfoliation. Structural correlations in the solvent indicate that shock induces an ice VII like motif in the first solvation shell of water.

## Introduction

Liquid-phase exfoliation (LPE)^1–7^ is a highly promising approach to large-scale production and dispersion of a wide variety of layered materials (LMs). It affords facile processing of individual nanosheets, which can be deposited on surfaces or combined into free-standing films^6^, and vertical or horizontal stacks. LPE has been used to create novel multi-ferroic materials for photoconducting cells^7–9^, p-n junctions, field-effect transistors^10,11^, and memory devices^12^ using stacks of layered perovskites. Integration of LMs using LPE has potential applications in large-area electronics^13^ and inkjet printing^14^.

A wide variety of transition metal dichalcogenides (TMDCs), metal oxides, and perovskites have been exfoliated into 2D layers by electrochemical, sonication and shear methods^1,6^. Here, we will focus on the sonication approach in which a bulk solid is suspended in a suitable solvent and exfoliated into atomically thin LMs by cavitation phenomenon. Since LMs are characterized by strong in-plane covalent bonds and weak out-of-plane interactions, it is possible to exfoliate LMs by weakening the interlayer van der Waals interaction using ultrasonic cavitation^2^. It is desirable to keep ultrasonic intensity low in order to avoid sonolysis and defects in 2D materials and to prevent radicals in solvents which may affect dispersion of LMs. Solvents play a critical role in efficient production of 2D materials by LPE. Experimental measurements^15,16^ of interfacial energy, Hildebrand solubility and Hansen parameter are commonly used to guide the selection of solvents for exfoliation of LMs. Solvents with weak volatility (*N,N*-dimethylformamide and *N*-methyl-2-pyrrolidone) and low boiling points (propanol, chloroform) have been successfully used to exfoliate LMs^1,15^.

Despite a great deal of experimental work, there is very little understanding of atomistic mechanisms underlying LPE. The motivation for the joint experimental and simulation work reported in the paper is to unveil the atomic mechanism of liquid-phase exfoliation and thus facilitate the synthesis of atomically thin layered materials (LMs). Shock exfoliation of LMs by bubble collapse mimics experimental conditions, and experimentalists are trying to optimize the conditions for liquid-phase exfoliation by choosing suitable solvents and shock intensity so that single sheets of defect-free LMs are produced. We have performed molecular dynamics (MD) simulations^17,18^ in which a MoS_2_ crystal is suspended in a solvent of water and isopropanol (IPA) containing a cavitation bubble. The system is subjected to a planar shock which initiates a chain of events in the solvent, culminating in the exfoliation of MoS_2_ into nanosheets. As the shock wave propagates through the solvent, the density of solvent increases to 1.6 g/cc and the number of nearest neighbors of a water molecule increases from 4 to 8, indicating an ice VII like motif. Water in the compressed solvent is not frozen: on the contrary, the self-diffusion coefficient of H_2_O molecules normal to the direction of shock propagation is increased by 60%. The shock wave impact collapses the cavitation bubble and generates a high-speed nanojet in the solvent. The nanojet impact generates large shear stresses (~10 GPa) on the MoS_2_ surface and the surface temperature goes up to ~3,000 K. These large shear stresses and elevated temperature initiate exfoliation of MoS_2_, and shock waves reflected from MoS_2_ surfaces enhance exfoliation. We have performed LPE experiment with IPA and DI water, which verify the conclusion from the simulations.

## Results

**Figure 1:**
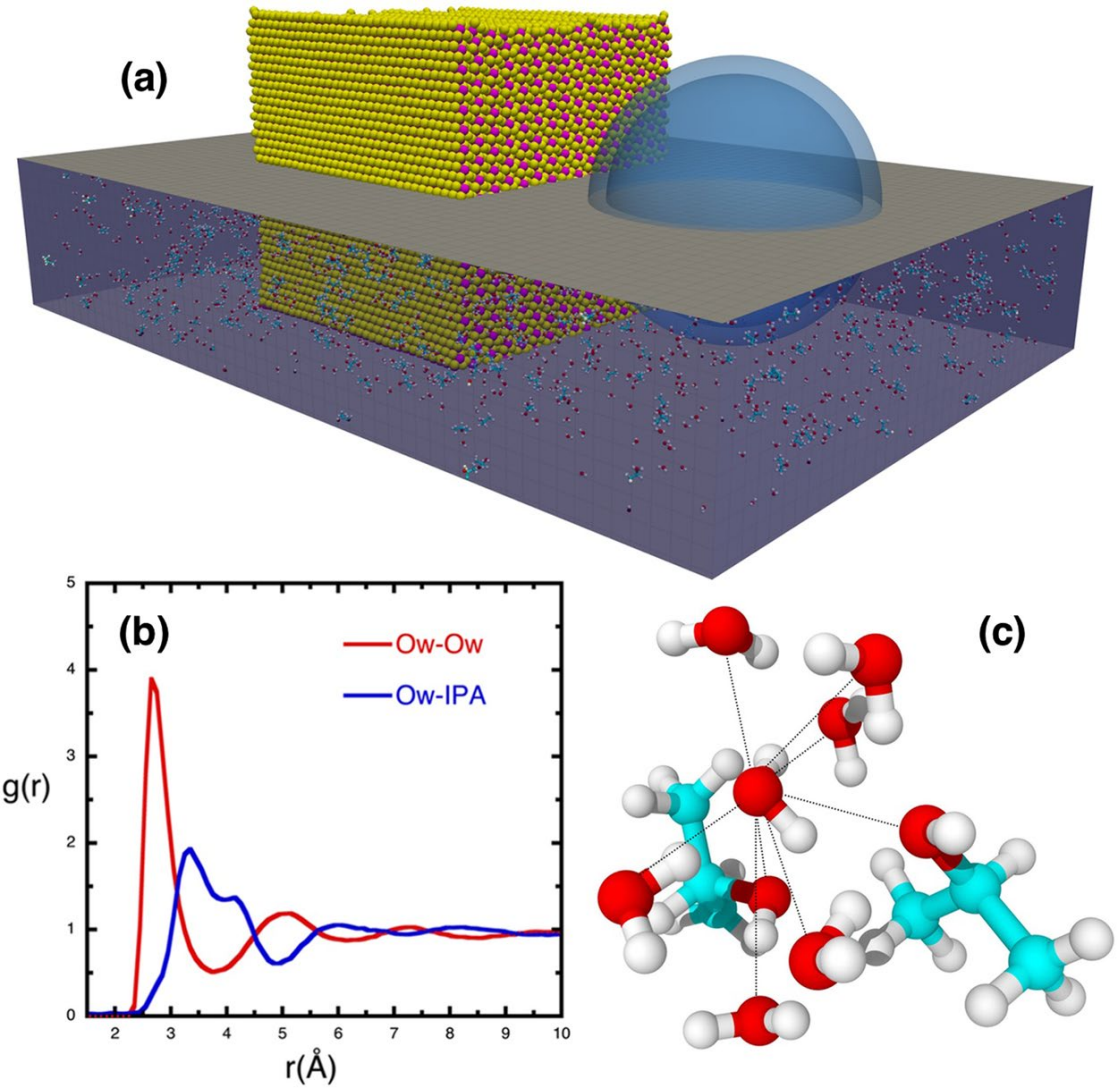
Initial configuration of the shock-induced exfoliation simulation and formation of ice VII motif. (**a**) Shows the initial setup of the exfoliation simulation. Bulk MoS_2_ is represented by pink (Mo) and yellow (S) sheets. MoS_2_ is immersed in a solvent, consisting of water and IPA molecules (1:1 ratio by weight). For clarity, only 2% of the solvent molecules (Oxygen: red, Carbon: cyan, Hydrogen: white) are shown in the lower half of the MD box. The solvent contains a nanobubble of radius *R* = 4.7 nm. The stand-off parameter, *d/R* = 1.14, where *d* is the distance between the bubble center and the closest MoS_2_ surface. (**b**) Shows the radial distribution function for oxygen-oxygen in water (red) and oxygen in water and the center of mass of IPA (blue). (**c**) Shows one water molecule with 8 nearest neighbors. Six of them are H_2_O and two are IPA molecules.

Figure 1(a) shows an initial configuration of a simulation in which an MoS_2_ solid (yellow and pink spheres) is immersed in a 1:1 mixture of H_2_O and IPA. (For the sake of clarity, only the lower half of the MD box is shown in the figure.) Cavitation is introduced after equilibrating the system under ambient conditions. The ratio of the bubble radius to the shortest distance between the bubble center and MoS_2_, *i.e*. the stand-off parameter *S*, plays a critical role in exfoliation. We performed simulations for several values of *S* ranging between 1 and 2 and observed exfoliation in the range 1.1 < *S* < 2.0 with particle velocity *V*_p_ = 3.0 km/s. Here we will present results for a bubble of diameter 9.4 nm and *S* = 1.14. Additional results are presented in the supplementary information.

In all simulations, shock is generated by a momentum mirror placed normal to the *z* direction just outside the MD box. The solvent, MoS_2_ and bubble are moved towards the momentum mirror with a constant speed *V*_p_ at time *t* = 0. When the solvent molecules cross the mirror, their momenta in the *z* direction are reversed which creates a planar shock wave in the solvent propagating away from the mirror. Using this approach, we first calculated the Hugoniot (shock-wave velocity *V*_s_ as a function of *V*_p_) of the solvent without MoS_2_ and found that it was similar to the Hugoniot of pure water; see Figure S2 in the Supplementary Information.

Shock simulations are performed at several particle velocities in the range of 0.5–4.0 km/s. Here we present results for the shock velocity *V*s = 7.4 km/s corresponding to the particle velocity *V*_p_ = 3.0 km/s. A movie of exfoliation is in the Supplementary Material. Under these conditions, the pressure in the solvent rises to 10.5 GPa and the density of water increases from 0.95 g/cc to 1.59 g/cc. This high compression has a dramatic effect on the structure of water. Figure 1(b) shows the radial distribution function *g*_o-o_(*r*) for oxygen atoms of water molecules. Here the first peak is located at 2.68 Å and the first minimum at 3.75 Å, whereas in pure water under ambient conditions the first peak and first minimum are at 2.76 Å and 3.34 Å, respectively. The average number of nearest neighbors of water molecules, calculated from the area under the first peak in Fig. 1(b) with a cutoff distance of 3.75 Å, is 8 as opposed to 4 in pure water under ambient conditions. The O-O-O bond-angle distribution for water molecules in the high-density solvent (HDS) peaks around 56^◦^ and the O-O-IPA distribution peaks around 65^◦^. These results indicate that the structure of water in the HDS is similar to that of ice VII in that both of them have the same density (1.6 g/cc) and number of nearest neighbors (8), see Fig. 1(c). However, the differences in bond-angle distributions reflect disorder in the first solvation shell of water in the HDS compared to the first solvation shell of ice VII^19,20^.

The MD results for the structure of water in the HDS are in good agreement with Dolan *et. al*.’s shock-wave experiment^20^ on pure water. They observed rapid freezing of water for *V*_p_ between 0.5 km/s and 2.0 km/s under isentropic and ramp-wave compression. Freezing occurred within a few nanoseconds above a critical value of pressure (7 GPa) irrespective of the peak pressure generated by shock. Their data clearly show that ultrafast homogenous nucleation of ice VII is feasible under shock compression above 7 GPA.

In the MD simulation, we do not observe complete freezing of H_2_O into ice VII because the timescale of the applied shock (~20 ps) is much shorter than the timescale (a few ns) in the experiment and also because of the presence of IPA molecules. However, we do observe a significant change in the dynamics of water molecules in the HDS: the self-diffusion coefficient of water, calculated from mean-square displacements normal to the direction of shock-wave propagation, is larger (3.7 × 10^−5^ cm^2^/s) than that of pure water under ambient conditions (2.4 × 10^−5^ cm^2^/s). The IPA molecules in the HDS diffuse much more rapidly than under ambient conditions: the self-diffusion coefficient for the center-of-mass motion of IPAs in the 50%wt mixture is 2.3 × 10^−5^ cm^2^/s in the HDS and 1.4 × 10^−6^ cm^2^/s under ambient conditions.

**Figure 2:**
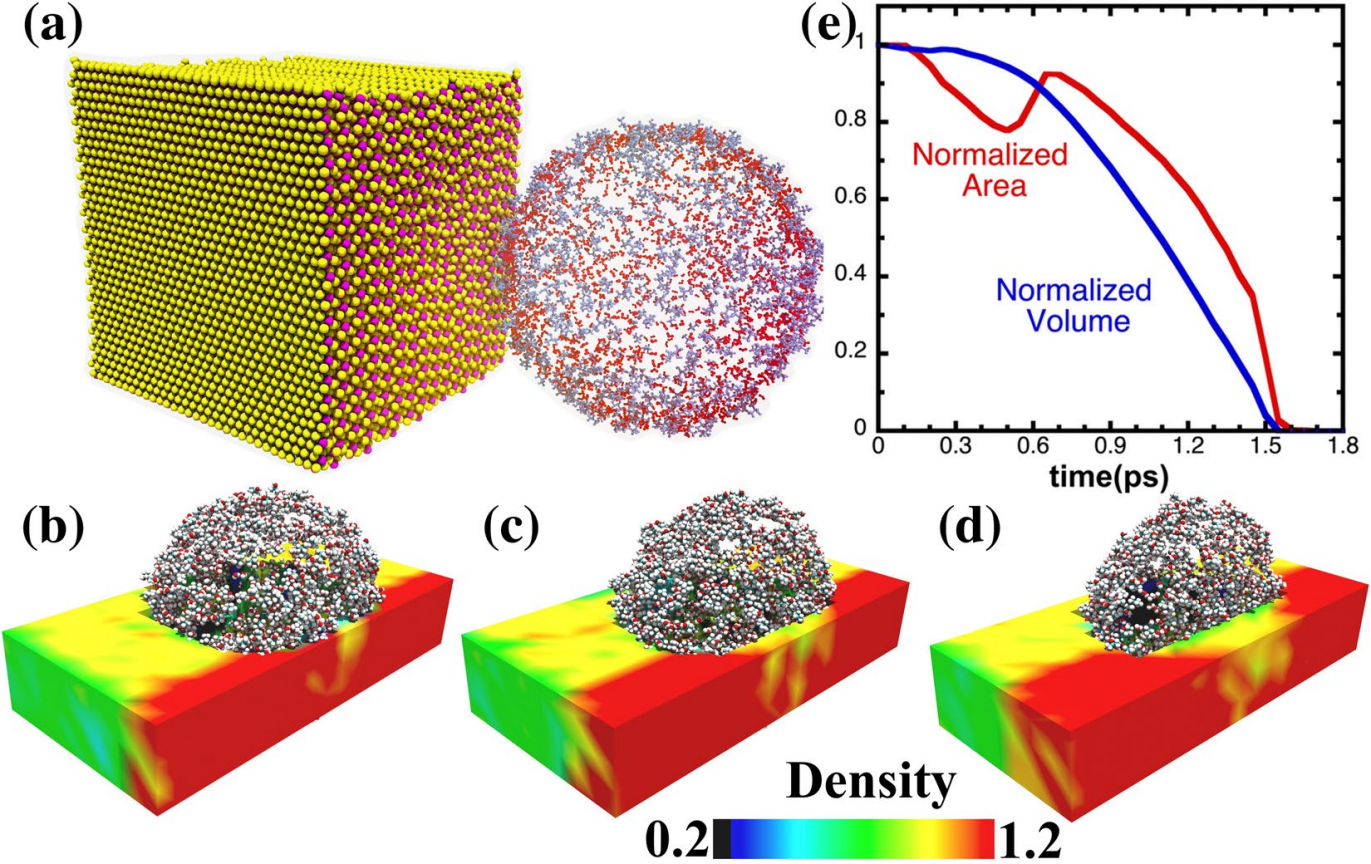
Snapshots of the collapsing cavitation bubble. (**a**) Shows the initial configuration of the cavitation bubble and MoS_2_. The bubble is represented by a shell of liquid molecules on the bubble surface. The standoff parameter is 1.14. At *t* = 0.2 ps, the shock wave hits the proximal side of the bubble. (**b**)–(**d**) Are snapshots showing changes in the shape of the collapsing bubble due to the shock wave at time *t* = 0.65, 0.85, 1.1 ps. The shock wave is represented by the change in the liquid density (half of the density is shown in the figures). (**e**) shows changes in the surface area and volume as a function of time while the bubble is collapsing. The surface area and volume are normalized to their respective initial values. The bubble collapses at *t* = 1.5 ps.

Under the impact of the shock wave, the bubble begins to shrink because the surface tension of the bubble cannot provide enough restoring force to balance the shock-wave compression. Snapshots in Fig. 2 show a time sequence of changes in the shape and size of the nanobubble resulting from the shock impact. As more and more solvent molecules enter the bubble, the proximal side of the bubble changes from spherical to ellipsoidal. The shape of the shock front also changes during bubble shrinkage: the front loses planarity because the solvent molecules entering the bubble have different velocities than the shock-front velocity *V*_s_. The front regains planarity after the bubble disappears. The bubble collapse time – the elapsed time between the onset of bubble shrinkage and complete bubble collapse – is 1.5 ps, see Fig. 2(b). It is in close agreement with the Rayleigh formula for bubble collapse time,

1 $$\begin{array}{c}\tau =0.45D\sqrt{\frac{\rho }{{\rm{\Delta }}P}}\,,\end{array}$$

where *D* is the initial diameter of the bubble, *ρ* is the fluid mass density (HDS in our case), and *ΔP* is the pressure difference across the bubble surface. Substituting *D* = 9.4 nm, *ρ* = 1.59 g/cc and *ΔP* = 10 GPa in Eq. (1), we find *τ* = 1.7 ps. It is remarkable that this estimate agrees so well with the MD result even though the effects of viscosity, surface tension and non-uniformity of the solvent near the bubble surface are ignored in the Rayleigh formula.

**Figure 3:**
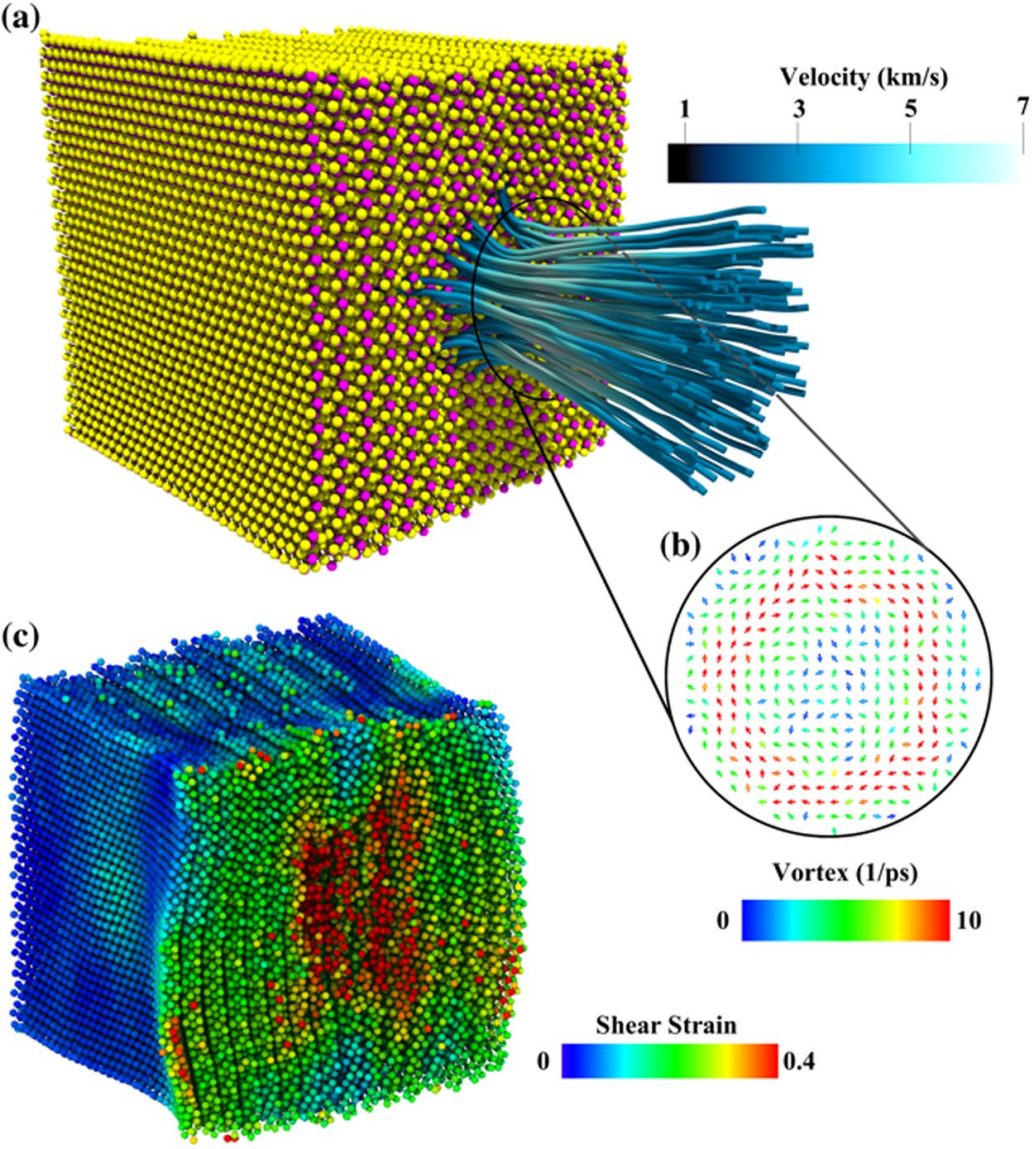
Shows cavitation bubble collapse giving rise to a nanojet and a vortex. (**a**) Snapshot taken at *t* = 1.75 ps shows velocity streamlines of the nanojet resulting from the bubble collapse. The color represents the magnitude of the stream velocity, which ranges between 3 and 6 km/s. The nanojet length and width are 6.5 nm and 3 nm, respectively, and the pressure at the tip of the nanojet is around 20 GPa. (**b**) The inset shows a vortex generated by the nanojet. The nanojet impact on MoS_2_ creates a 3 nm wide and 1 nm deep pit at *t* = 3 ps. (**c**) von Mises shear strain distribution in the pit region initiates exfoliation of MoS_2_.

At the onset of the bubble collapse, we notice sudden increases in the translational kinetic and rotational energies of solvent molecules at the shock front. These energy jumps are caused by solvent molecules entering the proximal side of the bubble. Velocity streamlines of these molecules are focused towards the bubble center in the form of a high-speed nanojet. Figure 3(a) shows the center-of-mass velocity streamlines of molecules in the nanojet at *t* = 1.75 ps after the bubble collapse. The nanojet length and width are 6.5 nm and 3 nm, respectively. At the tip of the nanojet, the velocity is 6.1 km/s and the pressure around 20 GPa, which is close to the pressure estimated from the jump condition, *P* − *P*_0_ = *ρ V*_p_*V*_s_ = 21 GPa. The nanojet length increases linearly with the initial diameter of the bubble^21^. Experiments^22,23^ show that this linear relationship also holds for micron-to-millimeter size bubbles. The nanojet generates a vortex (see the inset in Fig. 3(b)) whose angular velocity, calculated from the stream velocity $\overrightarrow{{\rm{\Omega }}}=\nabla \times \overrightarrow{{\rm{v}}}$, ranges between 5 and 15 ps^−1^. The nanojet hits the MoS_2_ surface at *t* = 1.75 ps after the bubble collapse. The impact causes pit formation on the MoS_2_ surface (indicated by the red region Fig. 3(c)), resulting in an 11% volume reduction at *t* = 3 ps. The pit is 3 nm wide and 1 nm deep. At *t* = 3.7 ps, the convex hull volume of MoS_2_ expands by 20%.

**Figure 4:**
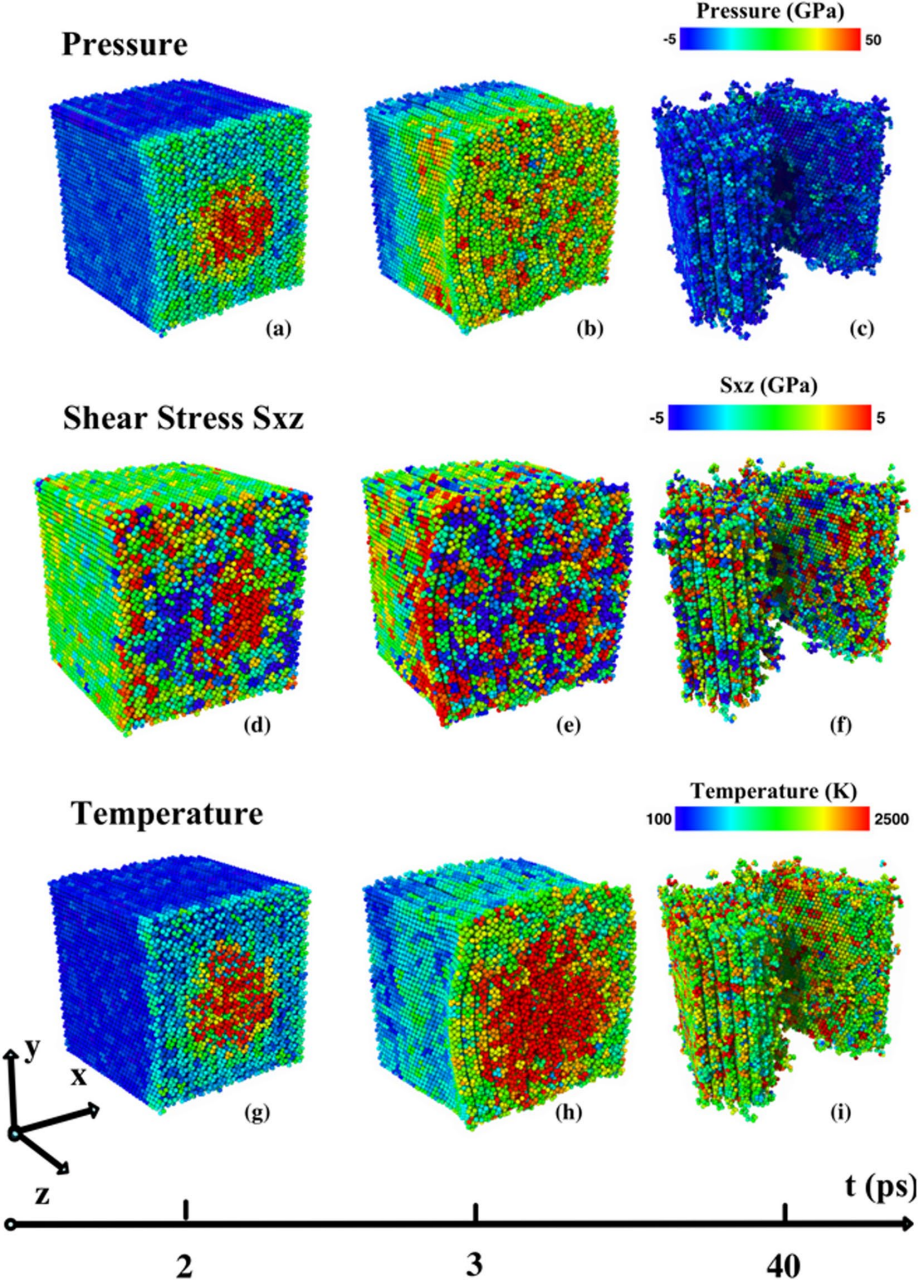
Pressure, temperature and shear stress distributions in MoS_2_ after the nanojet impact. (**a**) At *t* = 2 ps, the pressure is 40–50 GPa in the red region; 10–20 GPa in the green/yellow region; and around 0 GPa everywhere else. (**b**) Shows that the pressure is around 10–20 GPa over almost the entire MoS_2_ at *t* = 3 ps. (**c**) Shows that the pressure is released at *t* = 40 ps. (**d**) Shows the antisymmetric distribution of shear stress *S*_xz_ at *t* = 2 ps, (−10 to −5 GPa in the blue region, and 5~10 GPa at the center). (**e**) Shows *S*_xz_ spreads to initiate exfoliation at *t* = 3 ps. (**f**) Shows that the shear stress is released at *t* = 40 ps. (**g**) Temperature in the pit (red) is around 3,000 K, whereas the rest of the sample is around 300 K. (**h**) shows that the high temperature region becomes 7 nm wide and 1.5 nm deep. Here the temperature in the green and yellow regions is around 1,500 K. (**i**) Shows that the temperature in MoS_2_ is uniform and around 1,600 K after exfoliation.

The nanojet impact has a dramatic effect on exfoliation of MoS_2_. The time evolution of exfoliation is related to pressure, shear stress and temperature distributions in MoS_2_, see Fig. 4. The kinetic energy imparted by the nanojet significantly increases the pressure in the pit region of the MoS_2_. At *t* = 2 ps, the instant pressure in the 4 nm wide and 1 nm deep pit (red region in Fig. 4(a)) varies between 40 and 50 GPa. The pressure in the green and yellow regions around the pit ranges between 10 and 20 GPa, whereas the rest of the MoS_2_ is still at zero pressure. The pressure begins to drop as the pit region expands to 7 nm in width and 4 nm in depth at *t* = 2.3 ps. At 3.0 ps the pressure is between 20 and 30 GPa in almost all of MoS_2_ (see Fig. 4(b)), and after *t* = 7 ps the pressure drops to 0 GPa. Figure 4(c) shows the pressure distribution after MoS_2_ exfoliation at *t* = 40 ps.

Temperature fluctuations closely follow changes in the pressure in MoS_2_. The local temperature (calculated with peculiar velocity) in the pit ranges between 2,500 and 3,500 K at time at *t* = 2 ps. Away from the pit, the MoS_2_ remains at room temperature (indicated by the blue region in Fig. 4(g)). At *t* = 3 ps, the pit region cools off slightly due to expansion and the temperature distribution ranges between 2,500 and 3,000 K. At *t* = 4 ps, the temperature in MoS_2_ drops to 1,000 K except at the surface impacted by the nanojet where the temperature is 1,500 K. The temperature fluctuations persist until the very end of exfoliation, see Fig. 4(i).

The nanojet impact also generates large shear stresses, which initiate exfoliation of MoS_2_ into nanosheets. Figure 4(d) shows that the shear stress component *S*_xz_ in the red (compressive) and blue (tensile) regions fluctuates between 5 and 10 GPa, whereas in the rest of the MoS_2_ sample the shear stress is negligible. (Note, the shear-stress distribution is antisymmetric along the central plane at *x* = 100 Å.) A similar pattern is observed in the shear stress component *S*_yz_, which is antisymmetric along the plane *y* = 100 Å (see Figure S2 in Supplementary Information). These large shear stresses initiate exfoliation of MoS_2_ layers. Figure 4(e) shows that bulk MoS_2_ has partially exfoliated and the shear stress has dropped to 5 GPa at *t* = 3 ps. The shear stress is released after exfoliation, see Fig. 4(f).

The exfoliation is significantly enhanced every time the shockwave propagates through MoS_2_; see Figure S3 in the Supplementary Information. The instantaneous temperature of MoS_2_ increases to 1,300 K during the first encounter with the shock wave between 1.75 and 3 ps. Subsequently, the convex hull volume increases by 20% as 7,500 H_2_O and 1,700 IPA molecules flow inside the galleries of MoS_2_. The temperature of the sample decreases to 1,100 K at *t* = 12 ps and about 73% of the solvent remains in the MoS_2_ layers. The shock wave is reflected from the back end of the MD box (*z* = 0) at *t* = 8 ps and when the release wave hits MoS_2_ at *t* = 12 ps, the temperature of MoS_2_ increases to 1,400 K and the solvent content in the galleries of MoS_2_ increases with the addition of 3,000 water and 800 IPA molecules. Eighty percent of the solvent remains between the MoS_2_ layers after the passage of the release wave. As the shock wave reaches the opposite end of the box (*z* = 28.7 nm) and reflected again at time *t* = 18 ps, the temperature of MoS_2_ increases to 1,650 K at *t* = 23 ps, and an additional 5,700 H_2_O and 1,500 IPA molecules enter the galleries of MoS_2_.

Figure S3 in Supplementary Information shows solvent molecules between MoS_2_ nanosheets at *t* = 40 ps. Only 10% of the molecules chosen randomly are shown here. There are 13,500 water and 3,300 IPA molecules inside the sample at *t* = 40 ps. The volume of the convex hull continues to increase as more nanosheets exfoliate, see Figure S3(b). The swelling parameter of MoS_2_ plateaus at 2.0. The number of solvent molecules in the galleries of the exfoliated nanosheets increases rapidly when shock waves hit the MoS_2_ at *t* = 1.75, 12, and 18 ps; see Figure S3(c). Some of these solvent molecules diffuse out of the galleries. Overall, there is a dynamic balance between the number of solvent molecules entering and leaving the galleries of MoS_2_ sheets. Figure S3(c) in the Supplementary Information shows that the number of water molecules flowing in and out of the MoS_2_ sheets is larger than the number of IPA molecules because MoS_2_ is hydrophobic and water diffuses more rapidly than IPA.

**Figure 5:**
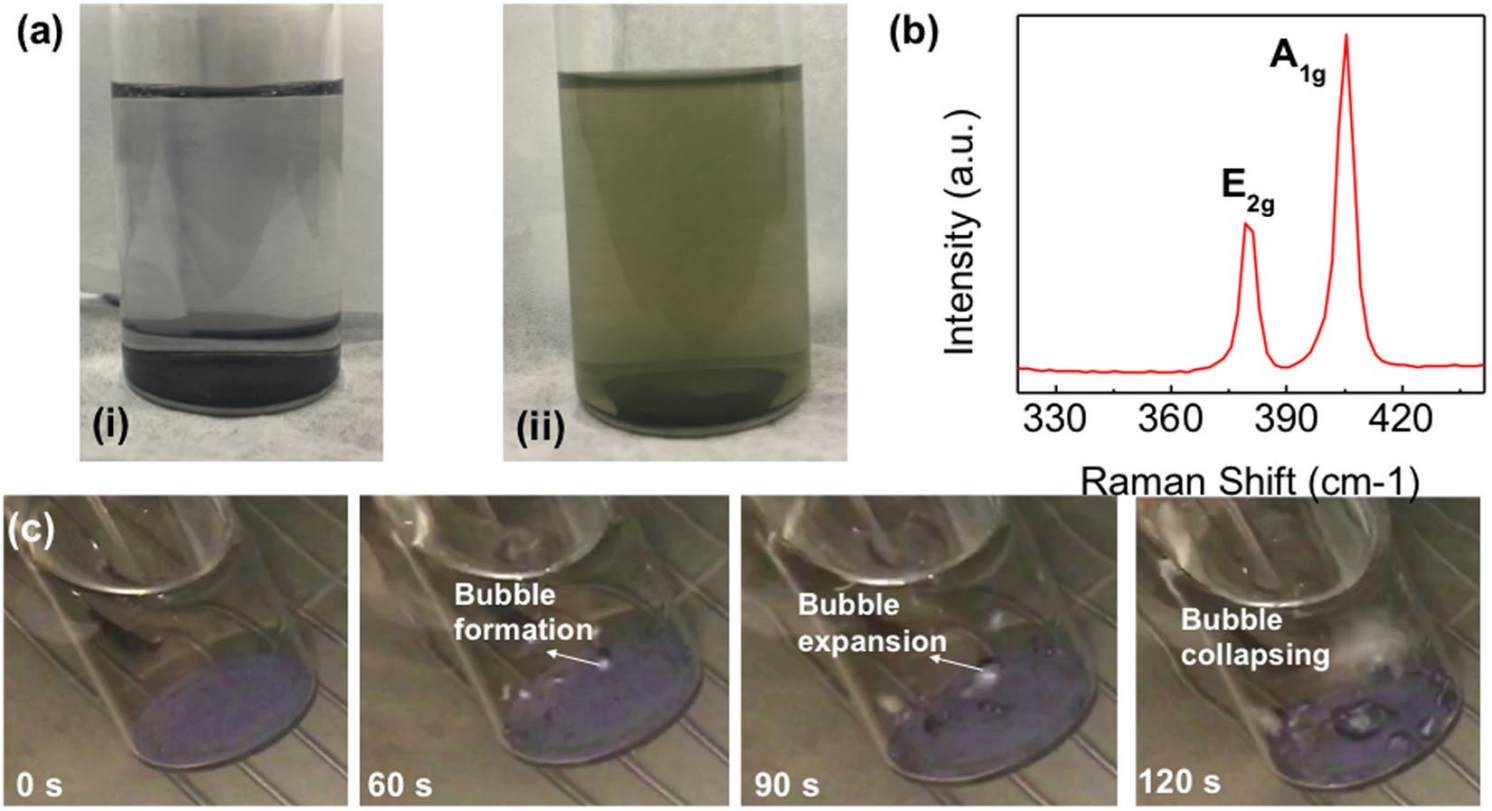
Exfoliated MoS_2_ with solvent between the nanosheets. (**a**) Commercial MoS_2_ in IPA + DI water solution (i) before and (ii) after exfoliation. (**b**) Raman spectrum of the exfoliated MoS_2_ flakes. (**c**) Snapshots of MoS_2_ flakes being exfoliated in IPA + DI water mixture. A small bubble formation takes place at the interface of MoS_2_ and solvent. The bubble expands after 90 s and collapses at 120 s, taking MoS_2_ powder along and dispersing it in the solvent. The color change in the liquid is an evidence of this phenomenon.

To verify the theoretical claims, experimental exfoliation of MoS_2_ was performed in IPA and DI water mixture (see Methods section). Figure 5(a) shows the black colored MoS_2_ powder in IPA + DI Water solvent before exfoliation. After exfoliation, the color of the solvent changes from transparent to light green due to the presence of 2D MoS_2_ flakes dispersed in the solvent. A slow-motion video was captured during exfoliation to examine different stages of exfoliation. Figure 5(c) shows snapshots of MoS_2_ powder at different stages of exfoliation. First, a bubble forms at the interface of MoS_2_ and solvent as a result of sonic wave propagation in the solvent. The bubble expands after 30 s and collapses after 60 s, resulting in shock wave inside the solvent and MoS_2_. The bubble also carries MoS_2_ with it, which is indicated by the local change in color of the solvent when the bubble bursts. The process repeats itself several times, resulting in uniform dispersion of 2D MoS_2_ sheets in the IPA + DI water solvent.

## Conclusion

In conclusion, MD simulations reveal atomistic processes underlying liquid-phase exfoliation (LPE) of LMs by sonication. Cavitation phenomenon underlies LPE experiments in which sonication probes are used to generate cavitation bubbles. We find that bubble collapse is a highly energetic process, and this is corroborated by experimental evidence of hot-spot formation in solvents^24^. Experiments^25^ indicate that temperatures in hot spots can be as high as 5,000 K. Our simulation shows that shock-induced bubble collapse results in the formation of a high-speed jet whose impact on an MoS_2_ surface initiates the exfoliation process. The nanojet impact raises the MoS_2_ surface temperature to 3,000 K and pressure to 20 GPa, and exerts a shear stress of 10 GPa. Exfoliation of MoS_2_ is initiated by this combination of large shear stress and temperature increase and, finally, exfoliation is significantly enhanced by repeated interactions of MoS_2_ with release waves resulting from the reflection of shock waves in the system.

The experiment has helped us validate the simulation. Experiments reveal that sonication causes cavitation and the collapse of cavitation bubbles generates shock waves in the solvent. Bubble creation and collapse are captured with high resolution camera and Raman spectra show that bulk MoS_2_ is exfoliated and flakes of MoS_2_ are observed. Our simulations are in accord with experimental observations. The simulation results - stress and temperature distributions, size and placement of bubbles, nature of solvent and solvent concentration - can help experimentalists with the optimization and scaling of exfoliation yield.

## Method

In the MD simulation, an MoS_2_ crystal of dimensions (9.8 nm)^3^ is suspended in an H_2_O/IPA mixture (1:1 ratio by weight) containing a spherical cavitation bubble of radius 4.7 nm. The system contains 10^6^ atoms and its dimensions are 19.7 nm × 19.7 nm × 28.7 nm in the *x*, *y*, and *z* directions, respectively. Periodic boundary conditions are applied along *x* and *y* and fixed boundary condition in the *z* direction. The simulation is carried out with a combination of force fields: TIP4P/2005^26^ for water, REBO potential^27,28^ for MoS_2_, and OPLS-AA force field^29^ for IPA. (The OPLS-AA is commonly used for organic molecules). The interaction between MoS_2_ and water is described by a combination of Lennard-Jones (LJ) and electrostatic potentials with force-field parameters taken from Luan *et al*.^30^, and we apply the Lorentz-Berthelot combination rule to parameterize LJ interactions between H_2_O, MoS_2_ and IPA molecules. Electrostatic forces and energy are calculated with the PPPM method, and slab correction^31^ is applied to allow for fixed boundaries in the *z* direction. Force fields are validated by experimental data on contact angles and surface tensions^15,32^, see the Supplementary Information Section 1. We use the Velocity-Verlet integrator with constraints on O-H bond and H-O-H bond angles of water molecules imposed with the SHAKE algorithm^33^. All simulations are done with open source package LAMMPS^18^ and the visualization is done with OVITO and ParaView^34,35^.

The system is relaxed for 250 ps at 300 K with a time step 0.5 fs and then a spherical nanobubble of radius 4.7 nm is created in the solvent. Exfoliation of MoS_2_ depends critically on the stand-off parameter, i.e., the ratio of the distance between the bubble center and the nearest MoS_2_ surface to the bubble diameter. The stand-off parameter was varied between 1 and 2 to determine the optimum value for exfoliation. The system was subjected to planar shock in the *z* direction using a momentum mirror. The particle velocity was varied between 0.5 and 4.0 km/s and the time step was reduced to 0.1 fs during shock.

To calculate thermal and mechanical properties in the system, the MD box is divided into 20 × 20 × 58 voxels and first the center-of-mass velocity of each voxel, ${\overrightarrow{v}}_{j}={\sum }^{}{m}_{k}{\overrightarrow{u}}_{k}/{\sum }^{}{m}_{k}$ is computed and subtracted from the velocity of each atom inside the voxel to get the thermal velocity ${\overrightarrow{v}}_{k,j}$. The instantaneous “temperature” distribution in the system is determined by calculating the kinetic energy of each voxel:

2 $$\begin{array}{c}\frac{1}{2}{N}_{f,j}{k}_{B}{T}_{j}=\sum _{k}\frac{1}{2}{m}_{k}{\overrightarrow{v}}_{k,j}^{2},\end{array}$$

where *k*_B_ is the Boltzmann’s constant, *m*_*k*_is the mass and ${\overrightarrow{v}}_{k,j}$ is the velocity of the *k*^th^ atom in the *j*^th^ voxel, and *N*_*f,j*_ is the number of degrees of freedom in the *j*^th^ voxel. The summation is over all the atoms in the voxel.

The pressure distribution in the system is calculated from the virial stress tensor for each voxel *j*:

3 $${S}_{j}^{\alpha \beta }=\frac{1}{V}\sum _{i\in V}[-{m}_{i}{v}_{ij}^{\alpha }{v}_{ij}^{\beta }+\frac{1}{2}\sum _{k}({r}_{kj}^{\alpha }-{r}_{ij}^{\alpha }){F}_{ik}^{\beta }],$$

where *V* is the volume of a voxel,$\,{r}_{ij}^{\alpha }$ and ${v}_{ij}^{\alpha }\,$are the Cartesian components of the position and velocity of the *i*^th^ atom in the *j*^th^ voxel, respectively, and ${F}_{ik}^{\beta }$ is the force on atom *i* due to atom *k*. The outer summation is over the atoms in a voxel, and the inner summation is over the atoms in the neighbor lists of atom *i*. The pressure in a voxel is given by,

4 $$\begin{array}{c}{P}_{j}=-\,\frac{1}{3}Tr({S}_{j}^{\alpha \beta }).\end{array}$$

We have also estimated the exfoliation yield by computing the accessible surface area^34,36^ of the MoS_2_ sample. The surface area is calculated with a sphere of radius 4.0 Å. To compute the swelling of the sample, a 3-dimensional convex hull^37^ is constructed with the largest clusters of MoS_2_ and the number of IPA and water molecules flowing in MoS_2_ galleries are counted.

To measure the impact on bulk MoS_2_, we calculate von Mises local shear strain^38^ ${\eta }_{k}^{{\rm{Mises}}}$ at each atom *k*. We choose the initial relaxed configuration of bulk MoS_2_ and the current configuration to get the local transformation matrix by minimizing

5 $$\begin{array}{c}\sum _{j}{|{\overrightarrow{r}}_{jk}^{0}{{\boldsymbol{J}}}_{k}-{\overrightarrow{r}}_{jk}^{1}|}^{2}\to {{\boldsymbol{J}}}_{k}={(\sum _{j}{\overrightarrow{r}}_{jk}^{0T}{\overrightarrow{r}}_{jk}^{0})}^{-1}(\sum _{j}{\overrightarrow{r}}_{jk}^{0T}{\overrightarrow{r}}_{jk}^{1}).\end{array}$$

Here the summation is over the nearest neighbors of atom *k*, ${\overrightarrow{r}}_{jk}^{0,1}\,$is the separation of atom *j* and *k* at the initial and current configurations. The shear strain of atom *k* is then computed as

6 $$\begin{array}{c}{\eta }_{k}^{{\rm{Mises}}}=\sqrt{{\eta }_{xy}^{2}+{\eta }_{yz}^{2}+{\eta }_{zx}^{2}+\frac{{({\eta }_{xx}-{\eta }_{yy})}^{2}+{({\eta }_{yy}-{\eta }_{zz})}^{2}+{({\eta }_{zz}-{\eta }_{xx})}^{2}}{6}},\end{array}$$

where *η*_*ab*_(*a*, *b* = *x*, *y*, *z*) are the six components of the local Lagrangian strain matrix **η**_*k*_ of atom *k*, and ${{\boldsymbol{\eta }}}_{k}=\frac{1}{2}({{\bf{J}}}_{{\rm{k}}}{{\bf{J}}}_{{\rm{k}}}^{{\rm{T}}}-{\bf{I}})$.

In the experiment, isopropanol (IPA) (99.99%; Sigma Aldrich) and de-ionised (DI) water were mixed in equal proportions. 2 mg of MoS_2_ powder (99.99%; Sigma Aldrich) was immersed in 50 ml of solvent mixture (IPA + DI water). MoS_2_ powder was allowed to sonicate for 48 hrs in the solvent. The temperature of the bath was maintained by changing the water bath every hour. Different stages of the exfoliation process were captured by a slow-motion video at 240 fps using a telephoto lens.

## Electronic supplementary material

Supplementary Information: Molecular Simulation of MoS2 Exfoliation

Supplementary movie

## Data Availability

All data generated and analyzed during this study are available in the paper and supplementary information. Extra data are available from the corresponding author on request.
