# Supplementary material for: Molecular Simulation of MoS2 Exfoliation
Source: Sci Rep. 2018 Nov 13;8:16761. doi: 10.1038/s41598-018-35008-z (PMC6233174; doi:10.1038/s41598-018-35008-z)
Supplement: Supplementary file 1 — Supplementary Information: Molecular Simulation of MoS2 Exfoliation [file 41598_2018_35008_MOESM1_ESM.docx]

**Supplementary Information:** **Molecular Simulation of MoS_2_ Exfoliation**

Guoqing Zhou^1^, Pankaj Rajak^2^, Sandhya Susarla^4^, Pulickel M. Ajayan^4^, Rajiv K. Kalia^1,2,3#^, Aiichiro Nakano^1,2,3^, Priya Vashishta^1,2,3^

^1^Collaboratory of Advanced Computing and Simulation, Department of Physics and Astronomy, University of Southern California

^2^Mork Family Department of Chemical Engineering and Materials Science, University of Southern California

^3^Department of Computer Science, University of Southern California

Los Angeles, California, 90089, USA

^4^Department of Materials Science and Nanoengineering, Rice University,

Houston, Texas 77005, USA

1. **Force-Field Validation by Experimental Measurement of Contact Angle**

The simulation reported here was performed with a combination of force fields: REBO (reactive bond order) for MoS2 and TIP4P/2005 for H_2_O. REBO potential accounts for changes in local atomic configurations of Mo and S, and TIP4P/2005 correctly models structural and dynamical properties of water. The interaction between MoS_2_ and water is described by a combination of Lennard-Jones (LJ) and electrostatic potentials with force-field parameters taken from Luan *et al*.^1^ We apply the Lorentz-Berthelot combination rule to parameterize LJ interactions between H_2_O, MoS_2_ and IPA molecules. Electrostatic forces and energy are calculated with the PPPM method, and slab correction^2^ is applied to allow for fixed boundaries in the *z* direction. We use the Velocity-Verlet integrator with constraints on O-H bond and H-O-H bond angle of water molecules imposed with the SHAKE algorithm^3^.

Force fields were validated by experimental data on contact angles and surface tensions^4,5^. According to Young’s law, this ensures the correctness of interfacial energy between the solvent and MoS_2_. The validation simulation was performed for a system consisting of a droplet of H_2_O/IPA mixture resting on an MoS_2_ substrate, see Figure-S1. We use free boundary conditions along *x* and *z* and periodic boundary condition in the *y* direction. The droplet of solvent molecules was put on top of a four-layer thick MoS_2_ substrate and the whole system was relaxed under ambient conditions until the contact angle had converged. The initial force field parameters between IPA and MoS_2_ were generated using the mixing rule. The energy parameter ε in the LJ force field was scaled by a parameter to fit two experimental measurements of the contact angle: one without IPA and the other for mass concentration of IPA around 50%. The final results are shown in the Table I.

| Mass fraction | MD (°) | Experiment (°) |
| --- | --- | --- |
| 0.0 | 101±4 | 97.8 |
| 0.035 | 75±3 | 70.6 |
| 0.16 | 39±2 | 44.8 |
| 0.36 | 31±4 | 31.1 |
| 0.56 | 19±1 | 24.8 |

Table I: Contact angles calculated by MD simulations for various mass fractions of IPA in the mixture. Experimental data are taken from Halim, U. *et al*^4^. After relaxing the system for 3 ns, six different configurations separated by 2 ps were used to calculate contact angles.


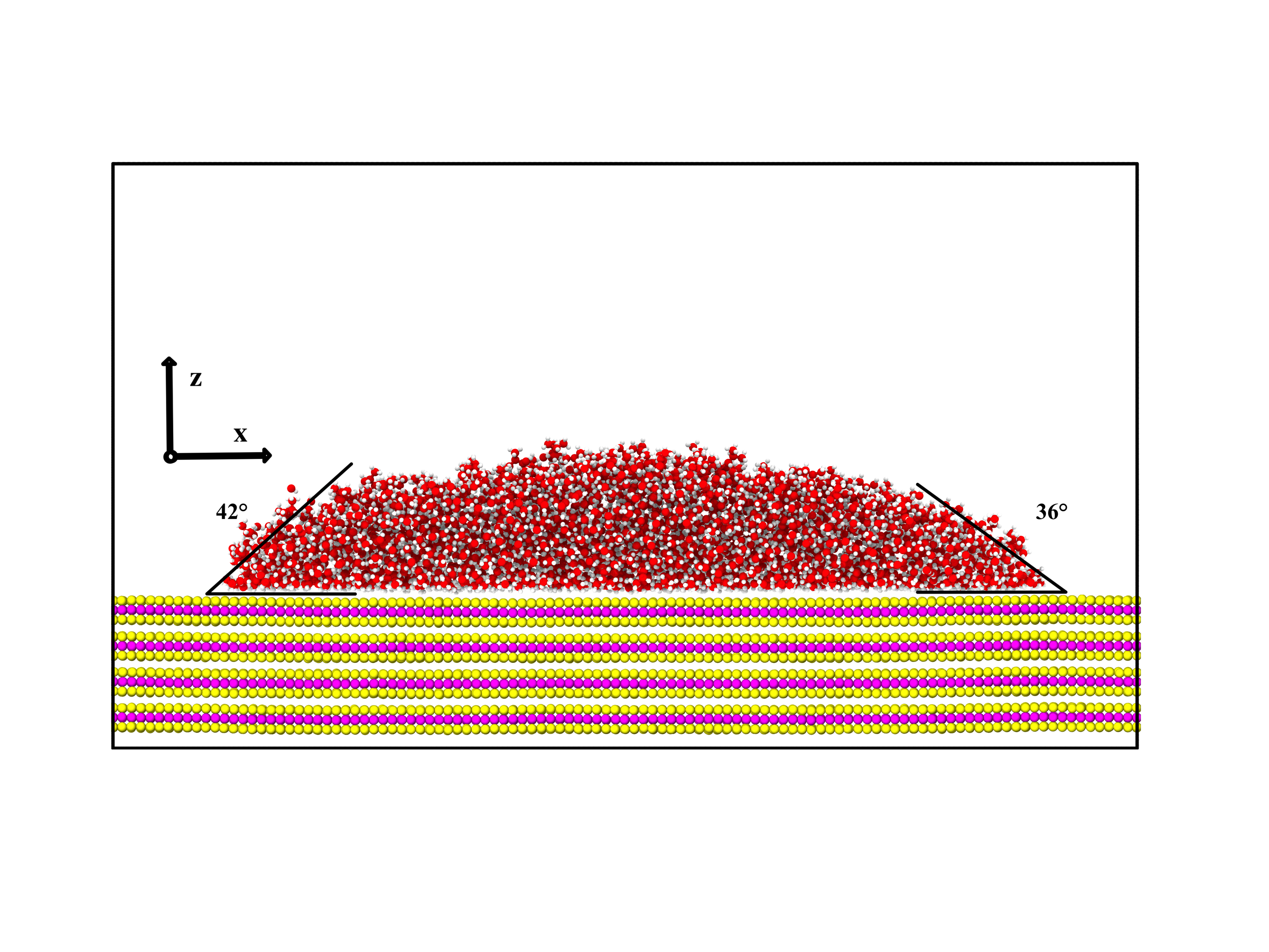


**Figure-1S:** Side view of the system for the calculation of contact angle of H_2_O/IPA (C, O: red, H: white) mixture on top of an MoS_2_ substrate (Mo: pink, S: yellow). Here the mass concentration of IPA is 36%. The calculated contact angle is 42° on the left and 36° on the right-hand side.

1. **Force-Field Validation for Shock Simulation**

To get the shock hugoniot of the H_2_O/IPA mixture, we apply different particle velocities *V*_p_ in our H_2_O/IPA mixture system to get the shock velocity *V*_s_. The system’s dimensions are 19.7nm×19.7nm×29.6nm in the *x*, *y*, and *z* directions, it contains 1.17 million atoms with 177332 water and 52974 IPA molecules (1:1 ratio by weight). The system is periodic along *x* and *y* directions while fixed along *z* with a momentum mirror on the ends of *z* direction. The system is first relaxed for 1 ns with timestep 2 fs under ambient condition. After that, a constant particle velocity *V*_p_ is added to the whole system. When atoms crossing the momentum mirror, they are reflected with their momenta in the *z* direction reversed, which generate a planer shock wave in the mixture propagating into the system. The shock velocity *V*_s_ is calculated with shock front boundaries at different time frames. The boundary is located with the abrupt of density. The simulation results show that the water/IPA mixture has similar shock hugoniot as pure water, see Figure-2S.


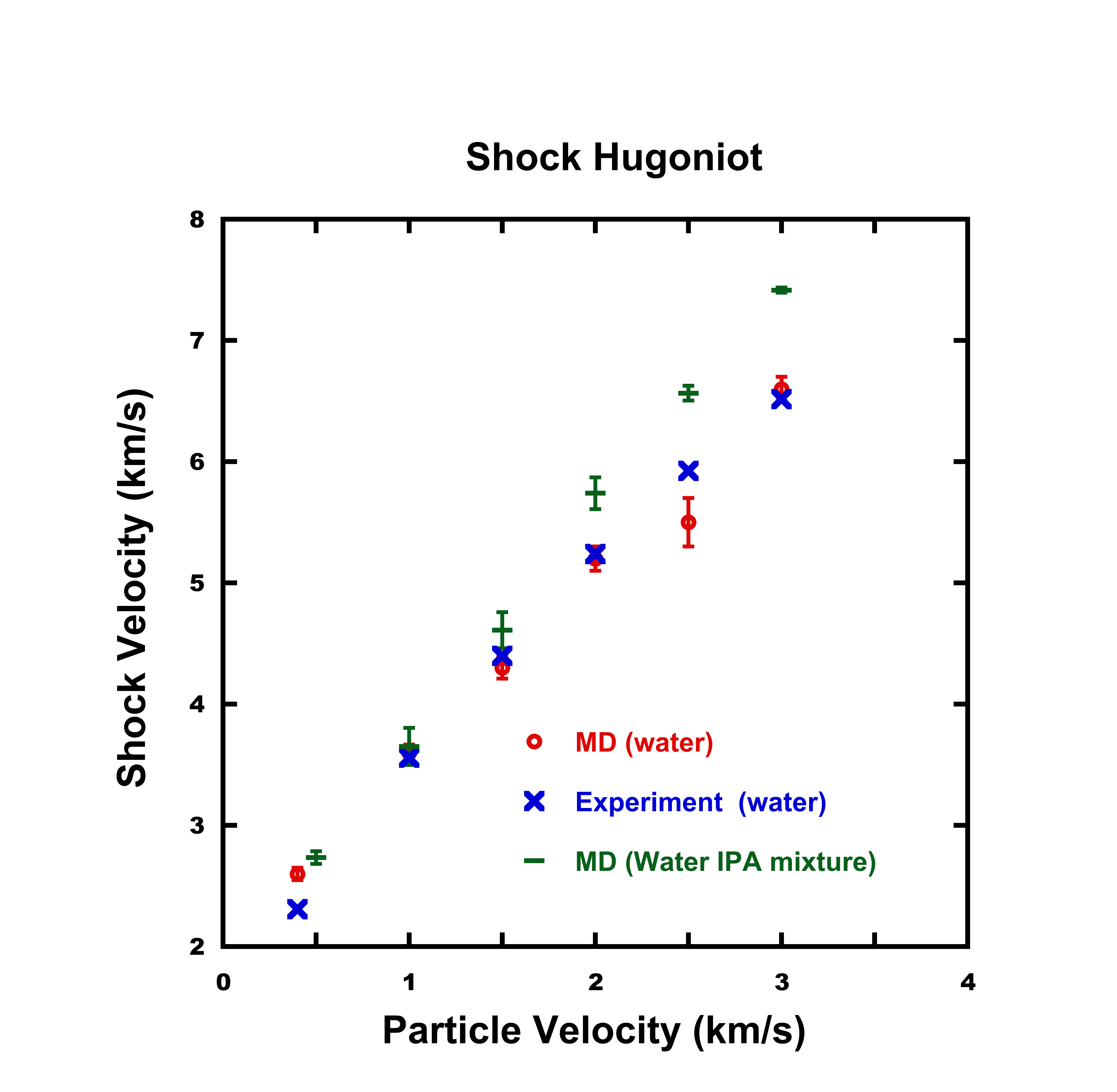


**Figure-S2** shows the Hugoniot compression curves of water (red circles: MD simulation, blue crosses: experiment) and water/IPA mixture (green segments: MD simulation) (50 wt% of IPA). The MD results and the experimental data for pure water are from Vedadi, M. *et al* ^6^

1. **Additional Results**

To quantify the exfoliation yield during the simulation, the accessible surface area^7,8^ and the volume of convex hull^9^ of the MoS_2_ bulk are calculated during the simulation as shown in Figure-S3(b). The convex hull of the bulk is used to count the number of solvent molecules flowing into and out of the MoS_2_ sheets during the shock wave propagation (see Figure-S3(a) and (c)). Each time when the shock wave hits (pass through) the bulk, solvent molecules are flowing into (out of) the sheets.


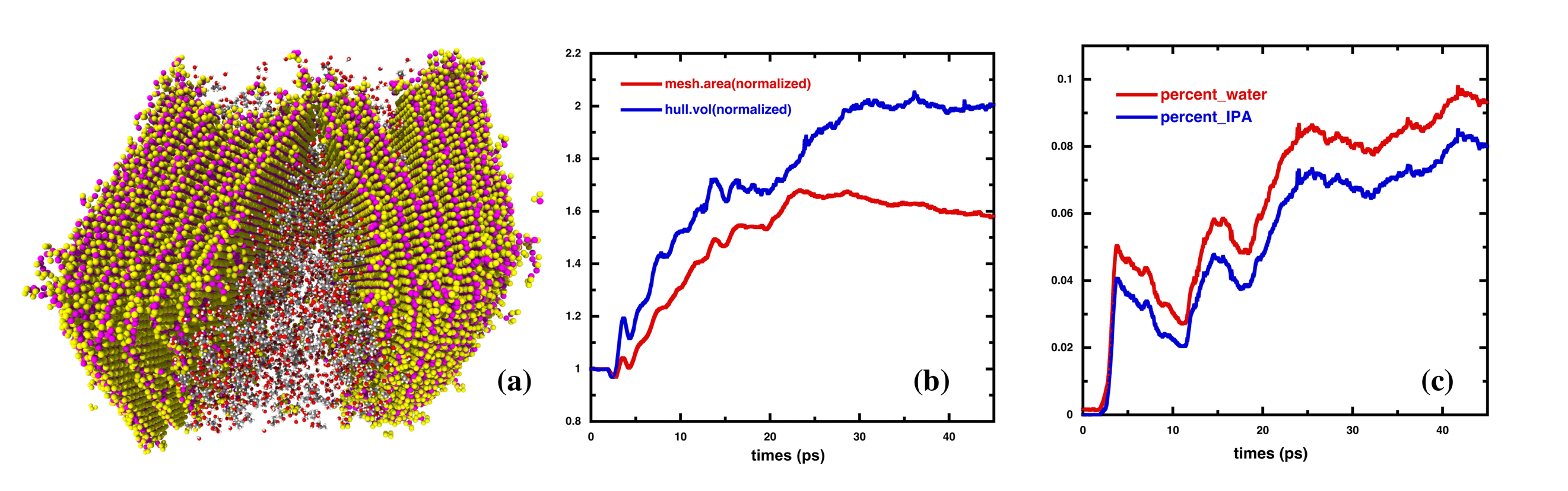


**Figure-3S: Exfoliated MoS_2_ with solvent between the nanosheets**. (a) Exfoliated MoS_2_ with only a fraction (10%) of solvent molecules shown in the figure at *t* = 40ps. (b) shows changes in the volume (blue line) and surface area (red line) of MoS_2_ during exfoliation as a function of time. The volume is that of the convex hull computed from the largest clusters of MoS_2_. The surface area is computed from the surface mesh of the bulk. Both the volume and surface area are normalized by their initial values. At the end of exfoliation the MoS_2_ volume expands by 100%, and the surface area increases by 60%. (c) shows the percentage of water (red line) and IPA (blue line) molecules in the convex hull as a function of time. Each time the shock wave hits MoS_2_ (at *t* = 2 ps, 12 ps, 18 ps), the solvent flows into the galleries of MoS_2_. After the shock wave passes, some of the solvent molecules flow out of the galleries.

Our simulations show that the exfoliation happens for standoff parameter *S* between 1.1 to 2.0. In Figure-S4 (a) we show exfoliation of MoS_2_ for *S* = 2.0. We performed simulations for particle velocities *V*_p_ = 0.5, 1.0, 2.0, 3.0, 4.0 km/s and did not observe exfoliation for *V*_p_ < 3 km/s. At *V*_p_ = 4.0 km/s, the nanojet impact disorders MoS_2_ and increases its temperature to 3,500 K. This indicates that the intensity of sonication plays a crucial role in exfoliation.

We also performed simulations at two different orientations of MoS_2_, one in which <0001> and the other in which <$2\bar{1}\bar{1}0$> surface of MoS_2_ is oriented to normal to shock propagation. At *V*_p_ = 3.0 km/s we find that the nanojet impact on the <0001> surface yields better exfoliation than the impact on the <$2\bar{1}\bar{1}0$> surface (see Figure-S4 (b)).


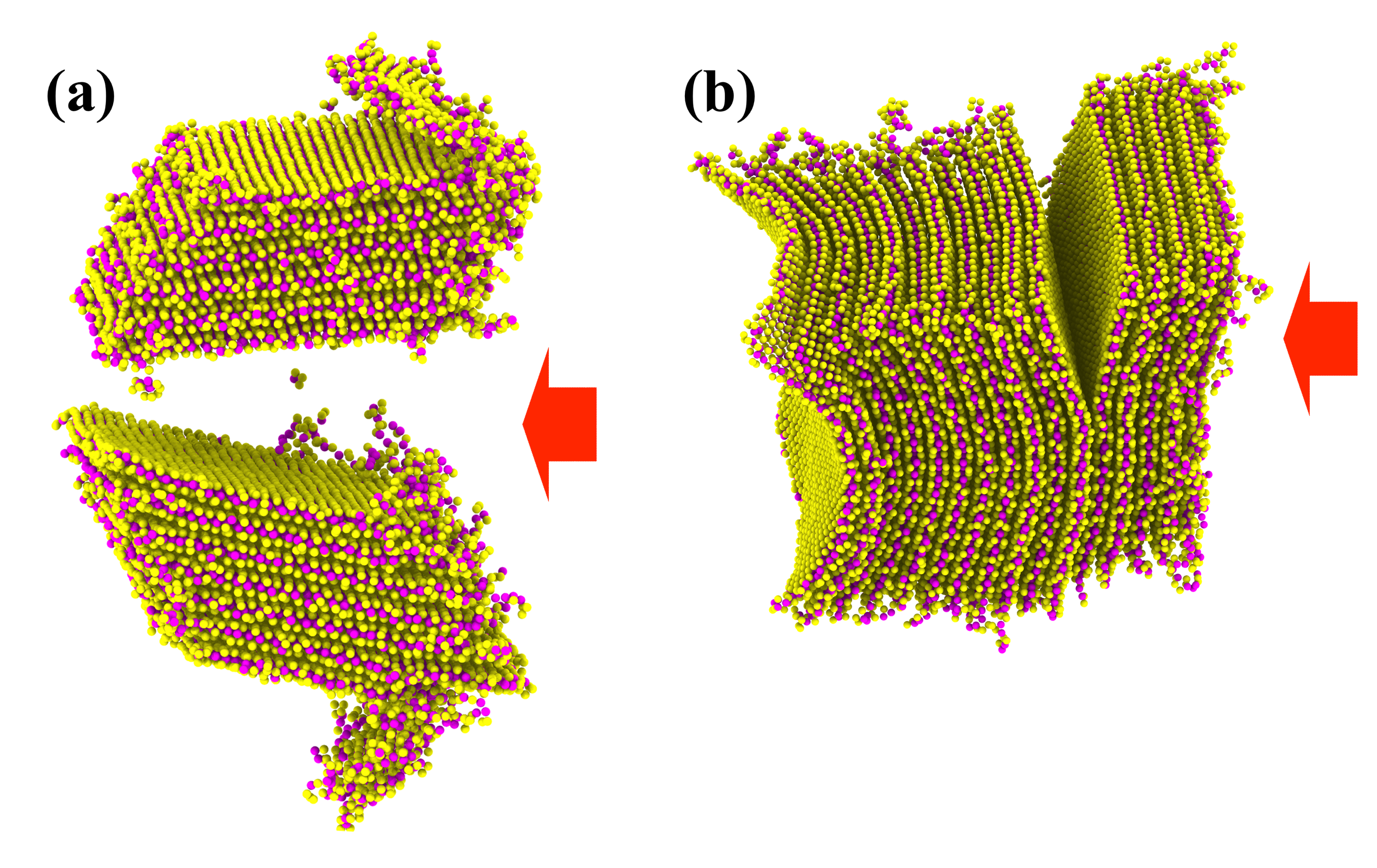


**Figure-S4** shows exfoliation of MoS_2_ samples (Mo: pink, S: yellow) in two MD simulations. (a) shows exfoliated MoS_2_ for *S* = 2.0, bubble size 5.0 nm, and particle velocity *V*_p_ 3.0 km/s when the shock is normal to the <$2\bar{1}\bar{1}0$> surface. (b) gives side view of cleavage in bulk MoS_2_ when the nanojet impacts the <0001> surface. In this case, the standoff parameter is 1.21, bubble size is 4.7 nm, and *V*_p_ is 3.0 km/s.

**References**

1. Luan, B. & Zhou, R. Wettability and friction of water on a MoS_2_ nanosheet. *Appl. Phys. Lett.* **108,** 131601 (2016).

2. Yeh, I.-C. & Berkowitz, M. L. Ewald summation for systems with slab geometry. *J. Chem. Phys.* **111,** 3155–3162 (1999).

3. Andersen, H. C. Rattle: A ‘velocity’ version of the shake algorithm for molecular dynamics calculations. *J. Comput. Phys.* **52,** 24–34 (1983).

4. Halim, U. *et al.* A rational design of cosolvent exfoliation of layered materials by directly probing liquid–solid interaction. **4,** 1–16 (2011).

5. Shen, J. *et al.* Liquid Phase Exfoliation of Two-Dimensional Materials by Directly Probing and Matching Surface Tension Components. *Nano Lett.* **15**, 5449-5454 (2015)

6. Vedadi, M. *et al.* Structure and dynamics of shock-induced nanobubble collapse in water. *Phys. Rev. Lett.* **105,** 2–5 (2010).

7. Stukowski, A. Visualization and analysis of atomistic simulation data with OVITO–the Open Visualization Tool. *Model. Simul. Mater. Sci. Eng.* **18,** 15012 (2010).

8. Stukowski, A. Computational analysis methods in atomistic modeling of crystals. *JOM* **66,** 399–407 (2014).

9. Barber, C. B., Dobkin, D. P. & Huhdanpaa, H. The quickhull algorithm for convex hulls. *ACM Trans. Math. Softw.* **22,** 469–483 (1996).
